# Adherence to Antiretroviral Therapy Among HIV-Infected Clients Attending Opioid Treatment Program Clinics in Dar es Salaam, Tanzania

**DOI:** 10.7759/cureus.25522

**Published:** 2022-05-31

**Authors:** John Kizindo, Alphonce I Marealle, Ritah Mutagonda, Hamu J Mlyuka, Wigilya P Mikomangwa, Manase Kilonzi, Raphael Z Sangeda

**Affiliations:** 1 Department of Clinical Pharmacy and Pharmacology, Muhimbili University of Health and Allied Sciences, Dar es Salaam, TZA; 2 Department of Pharmaceutical Microbiology, Muhimbili University of Health and Allied Sciences, Dar es Salaam, TZA

**Keywords:** integrated methadone and antiretroviral therapy, opioid treatment program, tanzania, pharmacy refill, self-report, adherence, antiretroviral therapy, hiv, methadone clinic, injectable drug users

## Abstract

Background

Adherence to antiretroviral therapy (ART) among key populations like human immunodeficiency virus (HIV)-positive People Who Inject Drugs (PWID) could be challenging, especially in low and middle-income countries (LMICs). Therefore we conducted this study to assess the adherence to ART among HIV-positive PWID attending three methadone clinics in Dar es Salaam, Tanzania.

Methods

A cross-sectional study was conducted at three methadone clinics in Dar es Salaam, Tanzania. Adherence to ART was measured by using pharmacy refill and patient self-report methods. Bivariate and multivariable logistic regression was performed to determine the association between dependent and independent variables. A p-value of less than 0.05 was considered to be statistically significant.

Results

Of the 180 participants, 97.2% recorded good adherence to ART as per the pharmacy refill method. However, only 66.1% of the PWID were found to adhere to ART based on the patient self-report method. Upon associating the self-report method with a viral load of >1000 copies/mL, participants were 3.37 times more likely to have missed their ART dose at least once in the last three days before their refill visit compared to those with a viral load of <1000 copies/mL [Adjusted Odds ratio; 3.37, 95% Confidence Interval (95% CI); 1.35 - 8.45, p = 0.009].

Conclusion

The adherence to ART among HIV-infected PWID attending methadone clinics was high based on the pharmacy refill method but relatively much lower based on the patient self-report method. There was a strong correlation between viral load and the level of adherence measured by the patient self-report method.

## Introduction

In 2020, the number of people infected with HIV in Eastern and Southern Africa was estimated at 20.6 million, with approximately 30% being People Who Inject Drugs (PWID) [1,2]. PWID are among the most vulnerable groups to HIV infection. A study conducted in Tanzania found that out of 480 people who used drugs, 13.5% of respondents injected drugs, of whom 67% shared needles suggesting that injection drug use may be a factor in the continuing epidemic in the country [3,4]. In 2015, the prevalence of HIV in PWID in Dar es Salaam, the largest city in Tanzania, was 15.5% which is three times that of the general population but relatively lower than in 2007, when the prevalence was estimated at 57% [5,6]. Despite the increased risk of HIV among PWID, they are also prone to have the least access to HIV prevention, treatment, and general health-care services because illicit drug use is often criminalized and stigmatized, affecting adherence to antiretroviral therapy (ART) [3]. For example, in Uganda, almost two-thirds (64%) of surveyed PWID said they avoided health-care services for fear of discrimination or being reported to law enforcement authorities [1].

To improve the management of PWID, the first Sub-Saharan Africa opioid treatment program (OTP) clinic using methadone was established in February 2011 at Muhimbili National Hospital (MNH) in Dar es Salaam, Tanzania. A second center in Tanzania was set at the Mwananyamala Regional Referral Hospital in 2012. The third clinic was at Temeke Regional Referral hospital in the same city. HIV care and treatment center (CTC) services were later incorporated into the methadone clinics to provide services to the HIV-infected PWID via an Integrated Methadone and Antiretroviral Therapy (IMAT) program [7]. It has been reported elsewhere that HIV-infected PWID who are on ART and enrolled in methadone clinics have improved adherence to ART, HIV-1 RNA suppression, and CD4 cell count response [8]. Various factors such as being homeless, female, and lower educational attainment have contributed to poor adherence to ART among PWID. Homelessness and low monthly income have also been associated with greater odds of HIV infection among PWID [3].

A study that compared people who do not inject drugs to PWID found that the latter had markedly lower plasma HIV-1 ribonucleic acid (RNA) suppression rates. Among patients who achieved any HIV-1 RNA suppression, PWID had dramatically higher rates of viral load rebound and these differences were primarily driven by lower levels of adherence to ART among the PWID [9]. A review that included fifteen studies from low and middle-income countries (LMICs) reported significant heterogeneity of ART adherence levels among HIV-infected PWID, which rules out generalization to the countries in similar economic groups [10]. More research is needed on ART adherence, given the critical importance of ART adherence among HIV-infected PWID in LMICs, including Tanzania. However, following CTC services incorporation in methadone clinics in Tanzania, there is a paucity of research on adherence to ART among HIV-infected PWID. Thus, this study aimed to determine the adherence level to ART among HIV-infected PWID on methadone therapy in Dar es Salaam, Tanzania.

## Materials and methods

Study setting and design

This descriptive cross-section study was conducted between April and June 2018 in all three methadone clinics in the Dar es Salaam region, Tanzania. Dar es Salaam is the largest city in Tanzania. In the 2012 Census, it was found to have a population of 4.36 million, accounting for 10 percent of the total Tanzania Mainland population [11]. It is also Tanzania's major business city. The first methadone clinic was launched at MNH in February 2011 and later at Mwananyamala and Temeke regional referral hospitals. As of December 2017, the MNH, which is the main clinic for PWID, had enrolled 1748 clients with 980 active clients [7]. This study was conducted at all three methadone clinics in Dar es Salaam.

Study population

The study population was HIV-infected PWID on ART attending methadone clinics in the Dar es Salaam region.

Sampling procedures

All HIV-infected PWID from the three methadone clinics in Dar es Salaam who were willing to participate in this study were enrolled. On the day of enrollment, the nurse counselor at the clinic approached the known HIV-positive PWID and asked for their consent to participate in this study. Before the interview was conducted, written prior informed consent was obtained from all participants.

Data collection

A questionnaire was employed in data collection whereby the first part gathered information on socio-demographic characteristics and selected clinical characteristics like CD4 cell counts and viral load levels. The second part of the questionnaire was designed to collect socio-demographic and adherence data. The questionnaire was administered to all participants. Data were collected and stored in the REDCap database [12,13].

Assessment of adherence

Adherence to ART was assessed using a modified Swahili version of the self-reported adherence assessment tool developed by the AIDS Clinical Trial Group (ACTG) [14]. PWID reported the number of missing doses on each day three days before the refill visit. Good adherence was reported when not a single dose was missed three days prior to the interview. A dose is the number of ART regimen pills required to be taken on a particular day as directed by the clinician.

Pharmacy refill information was obtained from facilities refilling date records. Each refill period was identified as the interval between the last visit and the scheduled new dates. Refill adherence was 100% if all pills had been picked up on time during the planned refill period. Refill percent values above 100 for patients who refilled earlier than scheduled were rounded to 100 percent. The calculation was based on the cumulative sum of the days that a patient was late for ART pick-up appointments in each month over the follow-up period, divided by the total number of days overall periods between pick-up periods in the follow-up period of study, resulting in the percentage of time the patient was without medication possession.

The calculation was made based on the following formula:

Adherence (%) = (number of days for the pills dispensed previously - delay in days for next pickup) / number of days for the pills dispensed previously) * 100

A subject was considered to have good adherence if scored a refill ≥85% and poor adherence if subjects had a refill <85%, the cut-off that was validated against viral load suppression in a previous study in the same setting [15].

Assessment of CD4 and viral load

Latest CD4 counts and viral load data were extracted from patients' medical records. According to HIV standard treatment guidelines in Tanzania, these measurements were taken within six months prior to the enrollment in the current study. The CD4 counts and viral load were used to measure treatment outcomes. Viral load success was defined as a viral load count below 1000 copies/ml [16] as per the National Guidelines for the Management of HIV and AIDS in Tanzania.

Data analysis

Data were entered using Microsoft Excel (Microsoft Corporation, Redmond, USA) and analysis was done using Statistical Package for Social Sciences (SPSS) version 22 (IBM Corp., Armonk, USA). Univariate analysis was carried out to determine the association between adherence as the dependent variable and independent variables. Variables with p < 0.05 were considered statistically significant in multiple logistic regression analysis.

Ethical consideration

Ethical clearance was obtained from the Muhimbili University of Health And Allied Sciences ethical committee. Permission to conduct the study in the selected sites was sought from the respective hospital management. The purpose of the study was fully explained to all participants, and written consent was obtained before the interviews. Confidentiality was ensured by using unique code numbers to hide the identity of participants.

## Results

A total of 180 PWID on ART were enrolled in the study, with the majority 60.0%, n = 109, from MNH. The mean age of participants was 37.6 years, ranging from 23 to 55 years old. The majority of the participants were male (62.2%). More than half (57.8%) of study participants were people with primary education levels. The ART regimen used by the majority (89.4%) of the study participants was a combination of tenofovir, lamivudine, and efavirenz (TLE) (Table 1). Other socio-demographic characteristics are summarized in Table 1.

**Table 1:** Social demographic and clinical characteristics of the study participants (N = 180) Key: TLE = tenofovir, lamivudine and efavirenz; CD4 = cluster of differentiation 4

Variable	Frequency (n)	Percentage (%)
Sex (n = 180)		
Male	112	62.2
Female	68	37.8
Age group <30 30-39 >40	20 90 70	11.1 50 38.
Education level (n = 180)		
No formal education	58	32.2
Primary Education	104	57.8
Secondary and tertiary education	18	10
Marital Status (n = 180)		
Married	81	45
Single	74	41.1
Divorced	25	13.9
Health facility (n = 180)		
Mwananyamala Regional Referral Hospital	59	32.8
Muhimbili National Hospital	109	60.6
Temeke Regional Referral Hospital	12	6.7
Type of antiretroviral therapy regimen taken (n = 180)		
TLE	161	89.4
Other regimens	19	10.6
Duration of antiretroviral therapy (n = 166)		
< 1year	9	5.4
1-3 years	89	53.6
>3 years	68	41.0
Viral load (copies/ml) (n = 108)		
<1000	73	67.6
>1000	35	32.4
CD4 cell count		
<200	13	17.6
>200	61	82.4

Level of adherence based on patient self-report and pharmacy refill methods

**Figure 1:**
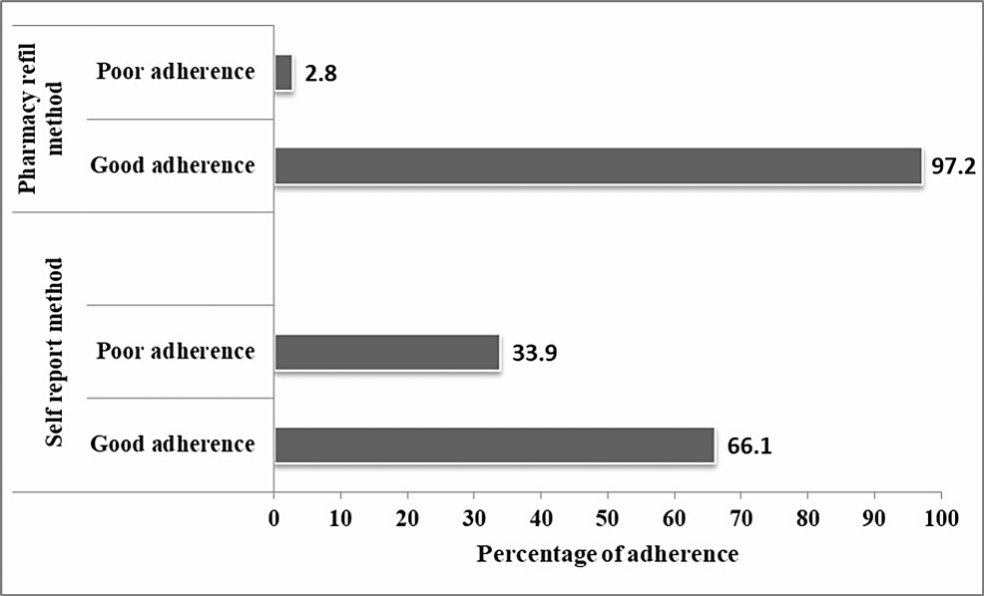
Level of adherence to antiretroviral therapy among HIV-infected PWID as measured by patient self-report and pharmacy refill methods (N =180) PWID: People Who Inject Drugs

Of the 180 study participants, 97.2% (n=175) had good adherence as per the pharmacy refill adherence measurement method. However, adherence measured by patient self-report methods was relatively lower compared to that of pharmacy refill method, whereby the proportion of participants with good adherence was 66.1% (n = 119). Figure 1 summarizes the level of adherence measured by both methods.

Reasons for missing ART

**Figure 2:**
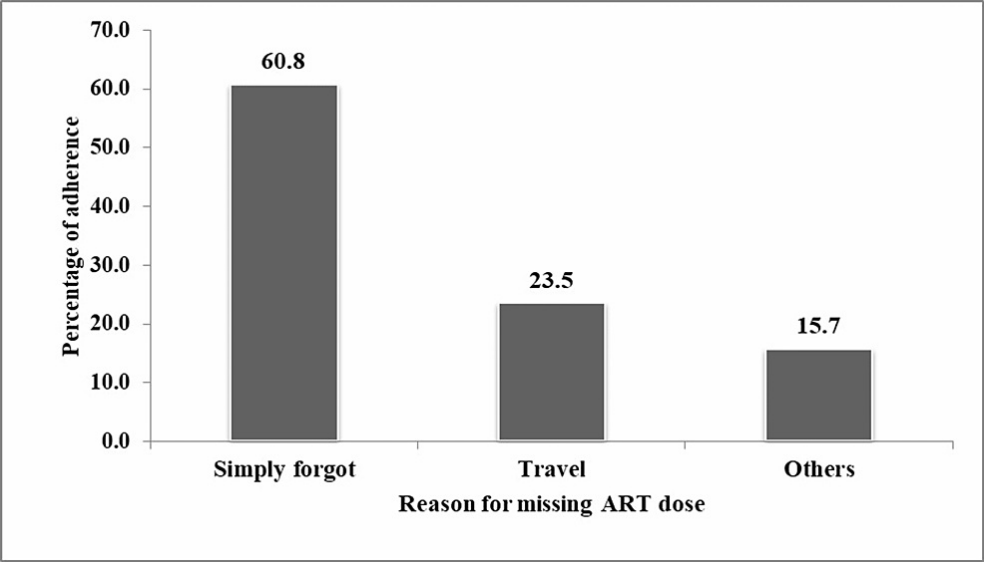
Reason for missing antiretroviral therapy in the last three days before the clinic visit (n = 51)

Among those who reported having missed a dose of ART in the last three days before a clinic visit, 51 (81%) provided the reasons for missing their dose (Figure 2).

The majority, 31 (60.8%), reported that they "simply forgot" to take their medication; 12 (23.3%) missed ART because they had traveled; the rest missed ART for other reasons, including a bad taste of the drugs, being arrested by police, watching football, being out of pills.

Given the lower proportion of adherence measured by patient self-report, further analysis was done to identify the possible predictors of adherence to ART (Table 2).

**Table 2:** Association between socio-demographic and clinical characteristics and adherence as measured by self-report Key: TLE = tenofovir, lamivudine and efavirenz

Variable	Good adherence n (%)	Poor adherence n (%)	Univariate analysis cOR (95% CI)	P-value	Multivariable analysis aOR (95% CI)	P-value
Age of the Subject (Years)						
<30	18 (90)	2 (10)	1		1	
30-39	59 (66.3)	30 (33.7)	4.58 (0.995 – 21.04)	0.051	11.28 (1.27 – 100.22)	0.03
≥40	41 (58.6)	29 (41.4)	6.37 (1.37 – 29.6)	0.018	11.92 (1.34 – 105.96)	0.026
Sex of the Subject						
Male	70 (62.5)	42 (37.5)	1.55 (0.81 – 2.97)	0.19		
Female	49 (72.1)	19 (27.9)	1			
Education Level						
No formal education	42 (72.4)	16 (27.6)	0.599 (0.198 – 1.814)	0.364		
Primary education	66 (63.5)	38 (36.5)	0.905 (0.324 – 2.53)	0.849		
Secondary to tertiary education	11 (61.1)	7 (38.9)	1			
Marital status						
Married	53 (65.4)	28 (34.6)	0.572 (0.231 – 1.42)	0.229		
Single	53 (71.6)	21 (28.4)	0.419 (0.169 – 1.092)	0.076		
Divorced	13 (52.0)	12 (48.0)	1			
CTC clinic attended						
Mwananyamala Regional Referral Hospital	42 (71.2)	17 (28.8)	0.81 (0.215 – 3.048)	0.755		
Muhimbili National Hospital	69 (63.3)	40 (36.7)	1.159 (0.328 – 4.095)	0.818		
Temeke Regional Referral Hospital	8 (66.7)	4 (33.3)	1			
Duration of antiretroviral therapy						
< 1year	5 (55.6)	4 (44.4)	2.06 (0.5 – 8.514)	0.317		
1-3 years	59 (66.3)	30 (33.7)	1.31 (0.659 – 2.61)	0.44		
>3 years	49 (72.1)	19 (27.9)	1			
CD4 cell count						
<200	10 (76.9)	3 (23.1)	1			
>200	35 (57.4)	26 (42.6)	2.48 (0.62 – 9.91)	0.2		
Viral load (copies/mL)						
<1000	56 (76.7)	17 (23.3)	1		1	
>1000	19 (54.3)	16 (45.7)	2.77 (1.18 – 6.55)	0.02	3.37 (1.35 – 8.45)	0.009
Type of antiretroviral therapy taken						
TLE	105 (65.2)	56 (34.8)	1			
Others	14 (73.7)	5 (26.3)	0.67 (0.229 – 1.955)	0.463		

There was a significant association between adherence levels measured by the patient self-report and viral load suggested with a p-value of 0.018 (Table 3). The pharmacy refill method was not significantly associated with virological outcomes in this PWID population (Table 3).

**Table 3:** Association of viral load and Pharmacy refill for 108 participants with viral load measurement

	Viral load (VL) category		
Adherence measurement method category	VL < 1000	VL ≥ 1000	p-value
	n (%)	n (%)	
1. Patient report			
Never Missed	56 (74.7%)	19 (25.3%)	
Missed	17 (51.5%)	16 (48.5%)	0.018
2. Pharmacy refill			
≥85	72 (67.3%)	35 (32.7%)	
<85	1 (100%)	0 (0)	0.487

## Discussion

To our knowledge, this is the first study in Tanzania to assess the level of adherence to ART among PWID since the incorporation of CTC services in the methadone clinics. In this study, the pharmacy refill method showed that almost all (97.2%) PWID had good adherence, while only 66.1% had good adherence to ART based on the self-report method. There was a significant association between adherence levels measured by the patient self-report and viral load suppression. The findings fall within the estimated range of PWID's level of adherence to ART of 33% to 97%, as reported by a systematic review study consisting of fifteen studies from seven countries using various methods of measuring adherence levels with the mean weighted adherence rate of 72% [10].

The difference in levels of adherence to ART observed in the two methods employed in this study can be attributed to the fact that PWID attend methadone clinics every day. Since CTC clinics are incorporated into the methadone clinics, the patients are unlikely to miss their ART refill dates. The high level of poor adherence observed by the self-report method (33.9%) may imply that most PWID adhere to the assigned pharmacy refill dates but might not take the medications as indicated. Additionally, by the time this study was conducted, there was compensation provided to the PWID when they attended their methadone clinics which might have driven better adherence to ART based on the pharmacy refill method.

The observed significant association between adherence levels measured by the patient self-report and viral load suggests that patient self-report could be a more reliable method for predicting virological outcomes in PWID over the pharmacy refill method. Similar findings have been reported elsewhere [17]. This finding contradicts a previous study where pharmacy refill was a significant predictor of virological failure among HIV adults on ART in a similar setting in Dar es Salaam. However, the later research was in a normal adult population. The PWID may be motivated to return for refills due to the need for methadone to alleviate addiction and thus inflating their pharmacy refill rates [15]. Identifying self-report as a predictor of virological outcomes among PWID is essential for healthcare providers and communities in Tanzania. The best method for measuring adherence would indeed be measuring drug plasma levels. Other advanced methods for measuring ART adherence include the medication event monitoring system (MEMS), which contains a cap fitted on medicine bottles to automatically record the time and date each moment the bottle is opened or closed. However, relatively cheaper and more rapid adherence measurement methods are needed in LMICs like Tanzania. Self-report adherence measure is convenient, valid, and could be a useful adherence measure to support the treatment of PWID living with HIV attending methadone clinics since they are vulnerable to falling out of care. While we recognize that obtaining viral load is essential to assessing the outcomes of care, the self-report could be helpful to clinicians in this situation, where it is often challenging to measure viral load regularly. Each of these adherence methods has its pitfalls. For instance, the participants' self-reported measure of ART adherence is subject to recall bias [15].

Unlike previous studies that reported a correlation between self-report adherence and CD4 counts [8,18], this study did not show a significant relationship. For example, only 74 (41%) of all 180 participants in this study had CD4 count reports in their files. Regarding viral load measurements, out of 180 participants in this study, 108 (60%) had a viral load measurement recorded in the past six months. The small number of HIV-infected PWID with a complete dataset found at the visited sites might have limited the power to find an association of ART adherence with CD4 counts and other patient factors.

In this study, younger age was negatively associated with adherence to ART, whereby participants who had less than 30 years had poor adherence compared to those above 30 years. This finding is congruent with other reports where young age has been reported to predict poor ART adherence and viral suppression among PWID [19].

This study found that some PWID missed ART doses in the last three days before their refill visit and the most common reason was that they "simply forgot" to take their medication, similar to a study conducted in Pakistan where the majority reported "simply forgot" as the reason for missing medicines in the last month [20]. In contrast, some studies were conducted in non-PWID, like one conducted in the USA, in which most of the patients missed ART over the last three days, with the main reason being running out of pills. In another study conducted in Uganda, running out of pills and feeling sick after taking medication were cited as the main reasons for missing medications [21,22].

Our study may be limited due to using two ART adherence measures among the other methods such as pill count, plasma concentration measurement, and electronic monitoring systems. The study may also be limited by the number of days the patient was followed. Pharmacy refills may best be studied by averaging refill periods over at least a year internal to capture missed refills over a more extended period [23]. Another limitation is the use of a viral load threshold of 1000 copies per ml as defined in the local Tanzania guidelines for the management of HIV. Nevertheless, the study pinpoints that pharmacy refill utility is limited in PWID's key population.

## Conclusions

The adherence to ART among HIV-infected PWID attending the methadone clinic was high based on the pharmacy refill method but relatively lower based on the patient self-report method. The level of adherence measured by patient self-report showed a significant correlation with the virological outcome. Therefore ART adherence level measured by patient self-report is likely to form a good predictor of virological outcomes in PWID attending methadone clinics in settings where PWID are enrolled in integrated Integrated methadone and antiretroviral therapy. These findings need to be emulated with a more rigorous follow-up study over a long period to be used to identify patients in need of adherence counseling.
